# Abiotic and biotic stresses induce a core transcriptome response in rice

**DOI:** 10.1038/s41598-019-42731-8

**Published:** 2019-04-18

**Authors:** Stephen P. Cohen, Jan E. Leach

**Affiliations:** 1 Department of Bioagricultural Sciences and Pest Management, Colorado State University, CO 80523-1177 Fort Collins, USA; 2 Cell and Molecular Biology Graduate Program, Colorado State University, CO 80523-1005 Fort Collins, USA

**Keywords:** Abiotic, Biotic

## Abstract

Environmental stresses greatly limit crop yield. With the increase in extreme weather events due to climate change and the constant pressure of diseases and pests, there is an urgent need to develop crop varieties that can tolerate multiple stresses. However, our knowledge of how plants broadly respond to stress is limited. Here, we explore the rice core stress response via meta-analysis of publicly available rice transcriptome data. Our results confirm that rice universally down-regulates photosynthesis in response to both abiotic and biotic stress. Rice also generally up-regulates hormone-responsive genes during stress response, most notably genes in the abscisic acid, jasmonic acid and salicylic acid pathways. We identified several promoter motifs that are likely involved in stress-responsive regulatory mechanisms in rice. With this work, we provide a list of candidate genes to study for improving rice stress tolerance in light of environmental stresses. This work also serves as a proof of concept to show that meta-analysis of diverse transcriptome data is a valid approach to develop robust hypotheses for how plants respond to stress.

## Introduction

Because plants are immobile, they must respond to and endure a wide variety of environmental and biotic stresses in the field. Both abiotic and biotic stresses cause major yield losses to crops^1–4^. It is therefore not surprising that many crop improvement programs focus on developing stress tolerant plant varieties^5–7^. Breeding tolerance for a single stress (e.g. drought, salinity, pathogen, etc.) or a single stress type (e.g. abiotic or biotic) may be risky because plants respond uniquely to different or simultaneous stresses, and increasing tolerance to one stress may be at the expense of tolerance to another^4,8^. With climate change, more extreme weather events are occurring, increasing the likelihood that plants experience multiple stresses in the field, including additional pressure from plant diseases^9^. There is, therefore, a need to understand the similarities and differences among stress response pathways to best optimize targeted crop improvement.

Plants respond to stress in a variety of ways. Common plant responses to avoid or tolerate abiotic stresses include stomatal closure, reduced photosynthesis, increased reactive oxygen scavenging activity, reduced leaf growth and increased root length^10^. Biotic stresses such as pathogens also cause plants to close stomata and reduce photosynthesis^11,12^. Other plant responses to pathogens include production of toxic compounds, including phytoalexins and reactive oxygen species, and induction of localized cell death^13^. Many of these responses are coordinated by phytohormones^14,15^. The hormones abscisic acid (ABA) and jasmonic acid (JA) are critical regulators of tolerance to abiotic stresses. For immunity to pathogens, plants primarily rely on salicylic acid (SA), JA and ethylene signaling. The abiotic stress response is regulated by many transcription factor (TF) families, both ABA-dependent and ABA-independent. The former includes ABA-induced basic leucine zipper (bZIP) TFs^16,17^. These TFs induce stomatal closure, expression of dehydration tolerance genes, and other adaptive physiological responses^18–22^. However, ABA often increases plant susceptibility in biotic interactions^23–27^ and frequently acts antagonistically with SA^28–30^.

With this study, we explore the rice transcriptome for a more thorough understanding of how rice regulates responses to multiple abiotic and biotic stresses. Previous studies have explored broad plant stress response by analyzing microarray data^31,32^. We expand on these studies with robust meta-analysis of publicly available rice RNA-Seq data sets. Our results reveal universally stress-regulated pathways, which we call the rice core stress response. The network of core stress-responsive genes presented here can be further explored for rice improvement in light of the need for tolerance to multiple environmental stresses. In addition to the valuable predictive transcriptome analysis for an important crop system, our approach can be easily expanded to other plant and crop systems.

## Results

### Meta-analysis of publicly available RNA-Seq data reveals the rice core stress response

To investigate the rice response to stress, we downloaded and analyzed publicly available RNA-Seq data sets representing rice transcriptome response to diverse abiotic and biotic stresses. These stresses include drought^33^, salt^34^, high and low temperature^35,36^, and infection with *Xanthomonas oryzae* pathovars *oryzicola* (Xoc) and *oryzae* (Xoo)^37,38^, *Magnaporthe oryzae*^39^, and Rice Stripe (RSV) and Dwarf (RDV) viruses^40,41^ (Table 1). All selected studies used stress-sensitive rice varieties. Four technical considerations were applied to choose RNA-Seq data sets: (1) there must be at least two replicates per treatment, (2) there must be untreated controls for each treatment, (3) tissue type was primarily above-ground, and (4) varieties were non-transgenic and non-mutant.

**Table 1:** Overview of NCBI SRA RNA-Seq accessions analyzed in this study.

Accession	Stress	Cultivar	Tissue	Time after stress	Replicates per Sample	Study Location
SRP071248	Drought long day Drought short day	Nipponbare	Leaf	13 d	3	Growth chamber
SRP052306	Drought	Nipponbare	Leaf	10 d	2	Greenhouse
SRP113286	Salt	9311	Seedling	1 h	3	Greenhouse
SRP101342	High Temperature	IRBB61	Leaf	6 h	2	Growth chamber
SRP004651	Cold	Nipponbare	Leaf	14 d	2	Growth chamber
SRP056884	*Xoc* BLS256 *Xoc* RS105 *Xoc* CFBP7331	Nipponbare	Leaf	2 d	3	Growth chamber
SRP049040	*Xoo* PXO349 1 dpi *Xoo* PXO349 2 dpi	Huanghuazhan	Leaf	1 d 2 d	3	Screenhouse
SRP076382	*M. oryzae* Guy11	Kasalath	Shoot	2 d	3	Unspecified
SRP049444	*M. oryzae* ZB13	Pid3	Leaf	1 d	2	Greenhouse
SRP065503	Rice Stripe Virus	Wuyujing 3	Leaf	7 d	3	Growth room
SRP115030	Rice Dwarf Virus	Zhonghua 11	Seedling	28 d	3	Greenhouse

Xoc indicates *X. oryzae* pv. *oryzicola*; Xoo indicates *X. oryzae* pv. *oryzae*.

**Figure 1:**
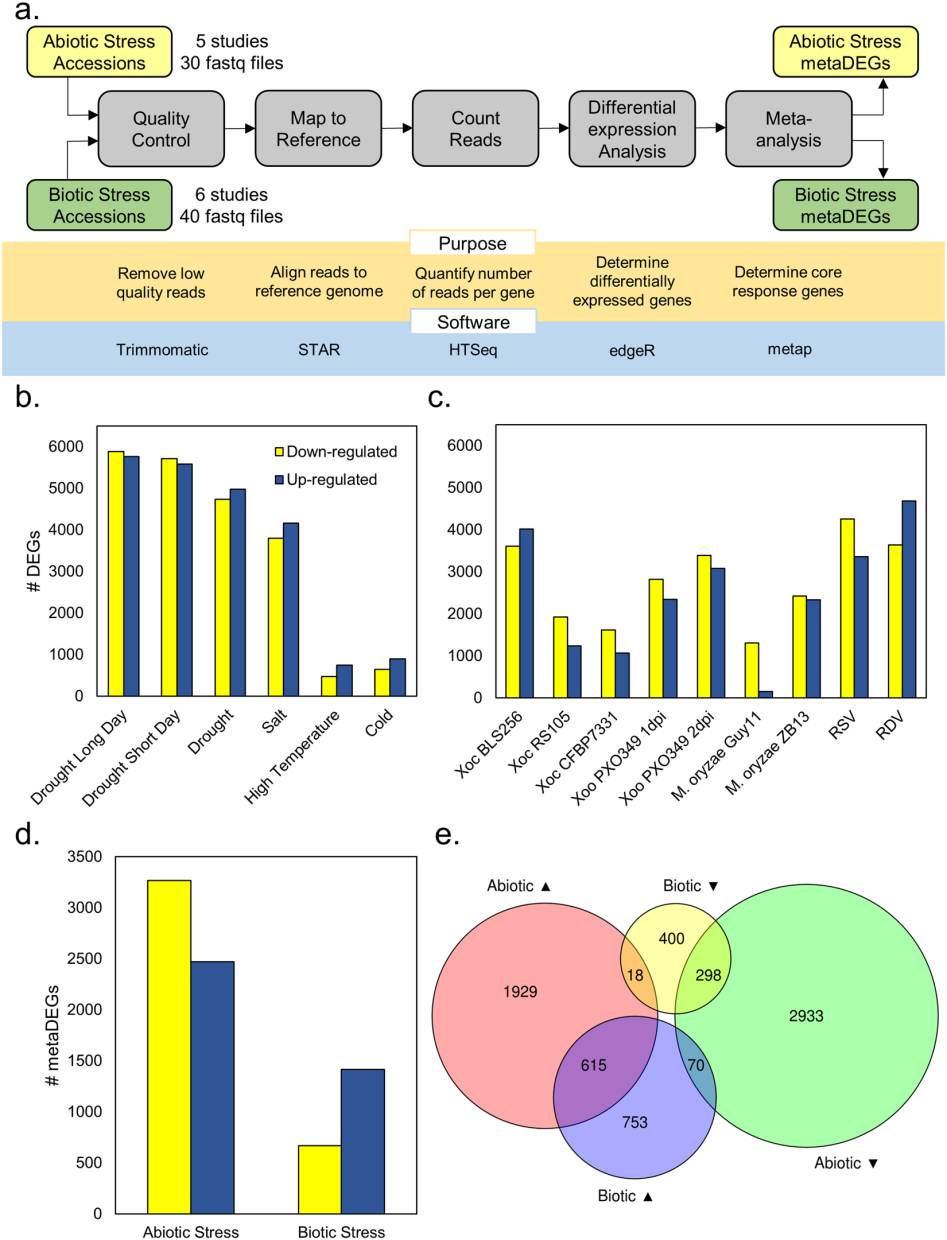
Analyses reveal rice core stress responses to abiotic and biotic stresses. (**a**) Analysis pipeline used to conduct differential gene expression analysis and meta-analysis on publicly available data sets. Number of DEGs identified in all (**b**) abiotic and (**c**) biotic stress experiments. (**d**) MetaDEGs identified from meta-analyses. (**e**) Number of metaDEGs unique and common in abiotic and biotic meta-analyses up- (up arrow) and down-regulated (down arrow).

A standard pipeline for consistently processing all raw sequencing data files was used (Fig. 1a). Included in this pipeline were steps for removing low quality reads, aligning to the reference genome, and counting reads. The proportions of reads mapped to the reference genome were generally high, with a mean of 77.4% total reads mapped to loci across all samples (Table S1). We conducted differential gene expression analysis separately on each experiment (Fig. 1a). The number of differentially expressed genes (DEGs) varied widely depending on stress treatment, and ranged from 1,220 to 11,644 DEGs (Fig. 1b,c, Table S2).

To explore the rice core response to abiotic stress, we used a meta-analysis to combine the results from all abiotic stress experiments (Fig. 1a). We found 5,863 meta-analysis-identified DEGs (metaDEGs) that were generally responsive to all abiotic stresses (Fig. 1d, Supplementary Data S1). We repeated this process to explore the core response to biotic stress, and found 2,154 metaDEGs generally responsive to all biotic stresses (Fig. 1d, Supplementary Data S1). Of the DEGs identified in the individual analyses, 10 to 43% were retained as metaDEGs (Table S2). The expression trends of the metaDEGs within individual studies followed the trends identified in the meta-analysis; that is, up- and down-regulated metaDEGs were mostly up- and down-regulated, respectively, within individual studies (Fig. S1). Therefore, this approach was valid for investigating rice core responses to abiotic and biotic stress.

To identify the rice response to all stresses, we investigated the overlap in expression patterns of the two sets of metaDEGs (Fig. 1e). We found all possible patterns of gene expression between abiotic and biotic stresses, including metaDEGs that were uniquely regulated by one stress type (abiotic or biotic), similarly regulated by both stress types, and oppositely regulated by both stress types (Fig. 1e, Table S3). Most metaDEGs were uniquely regulated by either abiotic or biotic stress. Interestingly, there were many more metaDEGs regulated similarly by both stress types (913 metaDEGs) than oppositely (88 metaDEGs). The annotations and median log_2_ fold changes of all 1,001 common metaDEGs are in Supplementary Data S2. Taken together, these results indicate there are: (1) genes responsive to a single stress type (abiotic or biotic), and (2) genes responsive to all stresses.

### Stress altered regulation of photosynthesis-related genes in rice

To investigate the rice biological processes (BP) altered during stress, we evaluated the enrichment patterns of the 45 BP gene ontology (GO) terms in abiotic and biotic up- and down-regulated metaDEGs (Table S4). The GO terms ‘catabolic process’, ‘cell communication’, ‘embryo development’, ‘reproduction’, and ‘response to extracellular stimulus’ were all enriched within metaDEGs (relative to background genes) up-regulated by both abiotic and biotic stress (Fisher’s exact test FDR-corrected p ≤ 0.01, Table 2). The GO terms ‘photosynthesis’, ‘protein modification process’, and ‘response to external stimulus’ were all enriched within metaDEGs down-regulated by both stresses. Several GO terms were enriched exclusively in abiotic or biotic metaDEGs, but no GO terms were enriched in genes oppositely regulated by stress type.

**Table 2:** Biological process GO terms exclusively enriched in up- or down-regulated metaDEGs.

GO Term	Abiotic	Biotic
**catabolic process**	Up	Up
**cell communication**	Up	Up
**embryo development**	Up	Up
**reproduction**	Up	Up
**response to extracellular stimulus**	Up	Up
**photosynthesis**	Down	Down
**protein modification process**	Down	Down
**response to external stimulus**	Down	Down
flower development	Up	*n.e*.
cell death	Down	Both
anatomical structure morphogenesis	Down	*n.e*.
cell differentiation	Down	*n.e*.
cell growth	Down	*n.e*.
cellular component organization	Down	*n.e*.
growth	Down	*n.e*.
ripening	Down	*n.e*.
tropism	Down	*n.e*.
multicellular organismal development	Both	Up
nucleic acid metabolic process	Both	Up
pollen-pistil interaction	Both	Up
post-embryonic development	Both	Up
response to endogenous stimulus	Both	Up
carbohydrate metabolic process	Both	Down

Terms indicated in bold are similarly enriched in both abiotic and biotic metaDEGs; Up, Down, and Both indicate terms are enriched in up-regulated, down-regulated, or both up- and down-regulated metaDEGs respectively; *n.e*. = not enriched.

There were 85 metaDEGs annotated with the GO term ‘photosynthesis’. These metaDEGs were generally down-regulated in individual transcriptome studies (Fig. S2). Rice down-regulated photosynthesis-annotated metaDEGs in response to drought, heat, cold, Xoc, *M. oryzae*, and RDV. Conversely, salt and RSV did not regulate these metaDEGs, and, in the study used, Xoo up-regulated them. These results indicate that altered regulation of photosynthetic pathways is a common rice response to stress.

### Stress up-regulated rice phytohormone-induced genes

**Figure 2:**
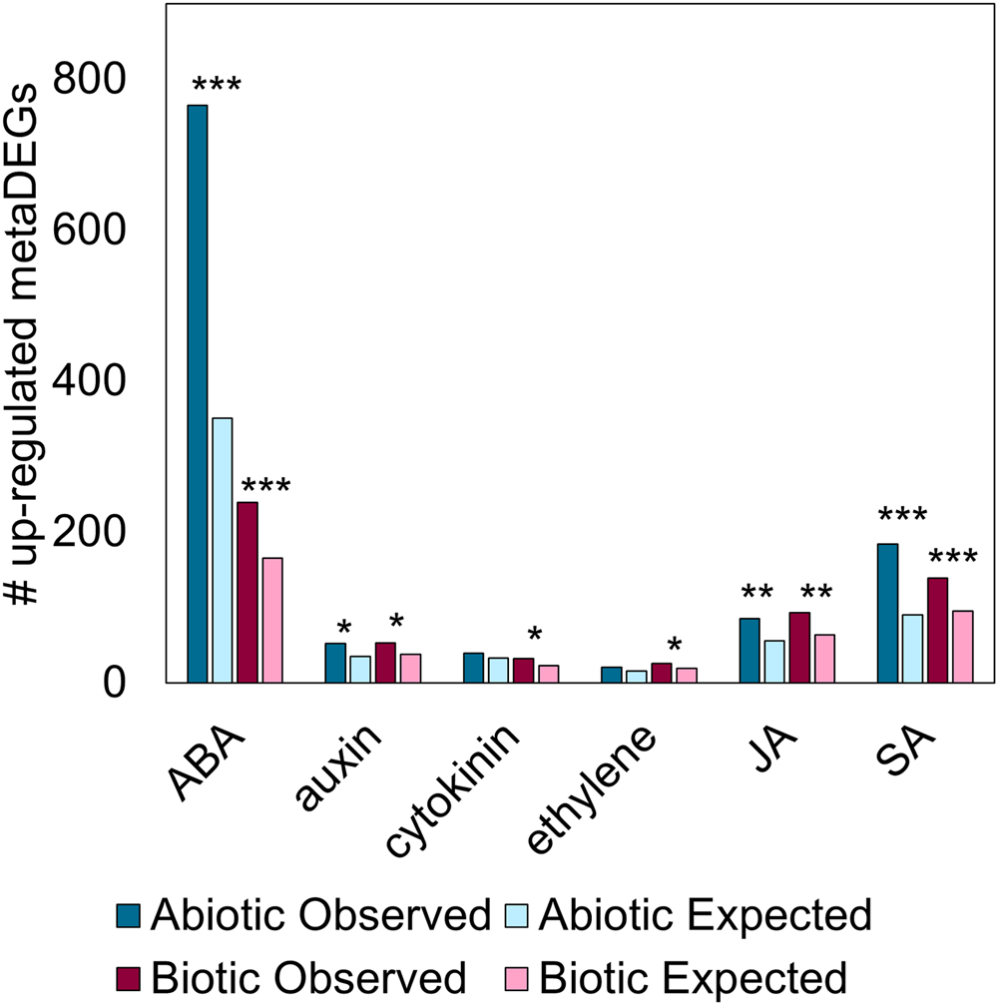
Rice hormone-responsive genes were generally up-regulated by stress. Observed number of up-regulated hormone-responsive metaDEGs is shown vs. the number expected to be up-regulated by random chance. Asterisks denote numbers observed differed significantly from numbers expected as determined by the χ^2^ goodness of fit test (***p < 10^−14^, **p < 10^−6^, *p < 0.005, see Table S5 for all p-values).

Because phytohormones are regulators of plant responses, we investigated how stress responses influenced phytohormone-induced genes. Abiotic metaDEGs responsive to ABA, auxin, JA and SA were more up-regulated than expected by random chance as determined by the χ^2^ goodness of fit test (p ≤ 0.05, Fig. 2, Table S5). Biotic metaDEGs in all hormone-responsive pathways were more up-regulated than expected. Response to ABA was the most significantly up-regulated hormone pathway in both abiotic and biotic metaDEGs, indicating that ABA signaling is likely important to the core stress response.

**Figure 3:**
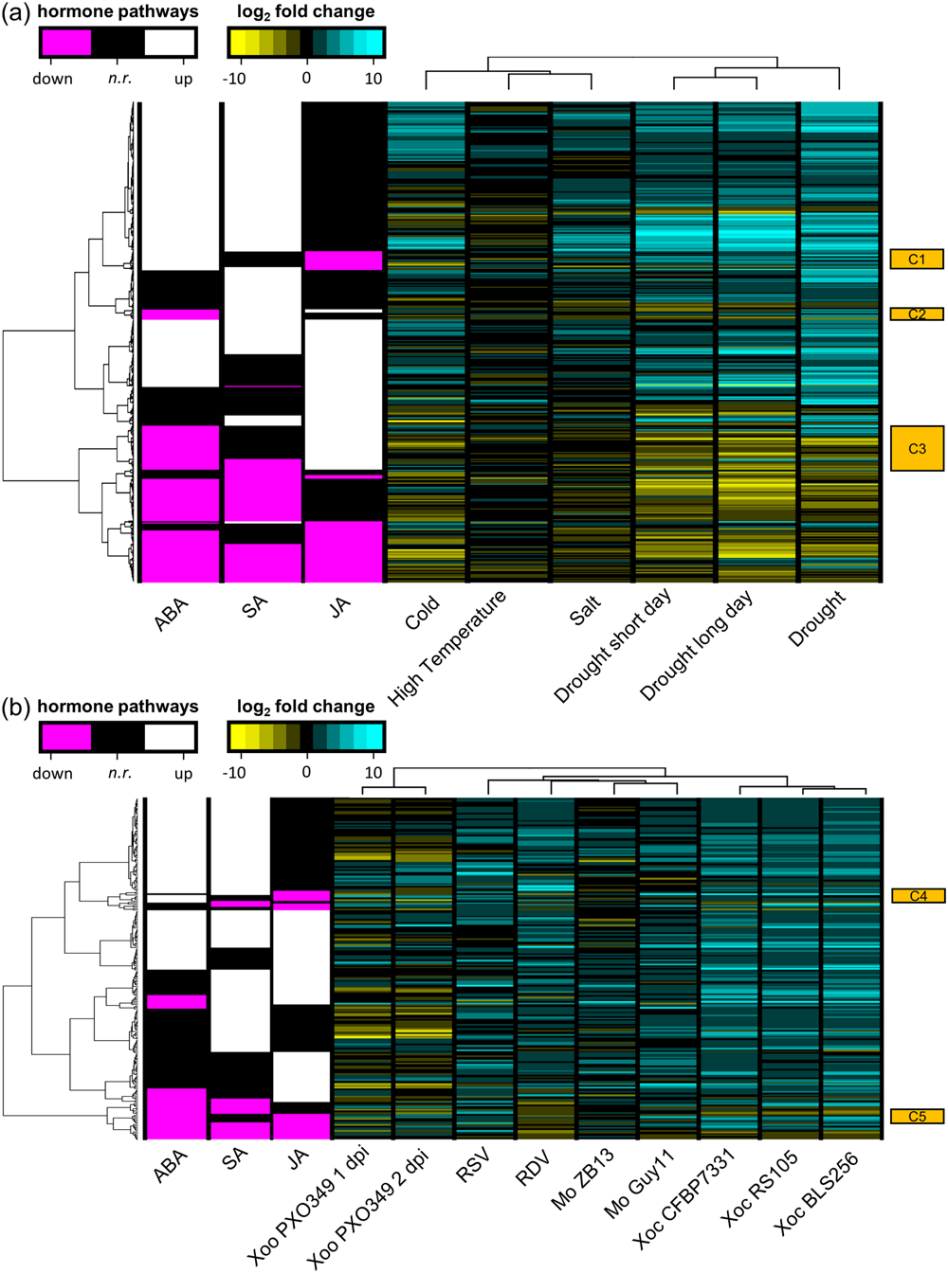
Signaling downstream of JA and SA is increased during stress. Gene expression (log_2_ fold changes) of JA- and SA-responsive metaDEGs for (**a**) abiotic stress and (**b**) biotic stresses relative to controls (columns) are shown on the right in yellow (down-regulated), black (not regulated) and cyan (up-regulated). Hormone regulatory patterns of JA- and SA-responsive metaDEGs are shown on the left in magenta (down-regulated), black (not regulated; *n.r*.) and white (up-regulated). Clusters of genes regulated oppositely of hormone pathways are indicated by the orange squares (C1 through C5).

There were 408 and 228 genes responsive to JA and/or SA in abiotic and biotic metaDEGs, respectively (Fig. 3, Supplementary Data S3). The expression of these genes indicate that during either stress type, JA and SA signaling are increased (Fig. 3). Only three small clusters identified within abiotic metaDEGs (Fig. 3a, labeled C1–C3) and two small clusters within biotic metaDEGs (Fig. 3b, labeled C4–C5) did not follow this trend; genes in these clusters were regulated oppositely by stress and hormones (JA and/or SA). Interestingly, in the Xoo study used for this validation, the expression of JA- and SA-responsive genes was generally opposite of all other biotic stress responses. JA and SA response were a larger component of the biotic stress response (10.6% of biotic metaDEGs) than of the abiotic stress response (7.0% of abiotic metaDEGs). Many of the JA- and/or SA-responsive genes were also responsive to ABA (Fig. 3). Genes responsive to JA and/or SA, but not responsive to ABA, were still up-regulated more than expected by random chance (Table S5). However, this was a much smaller proportion of metaDEGs (1.4 and 3.5% of abiotic and biotic metaDEGs, respectively). Taken together, these results indicate that in responses to any stress, rice orchestrates responses via phytohormones.

### Discovery of promoter motifs important to the stress response

**Figure 4:**
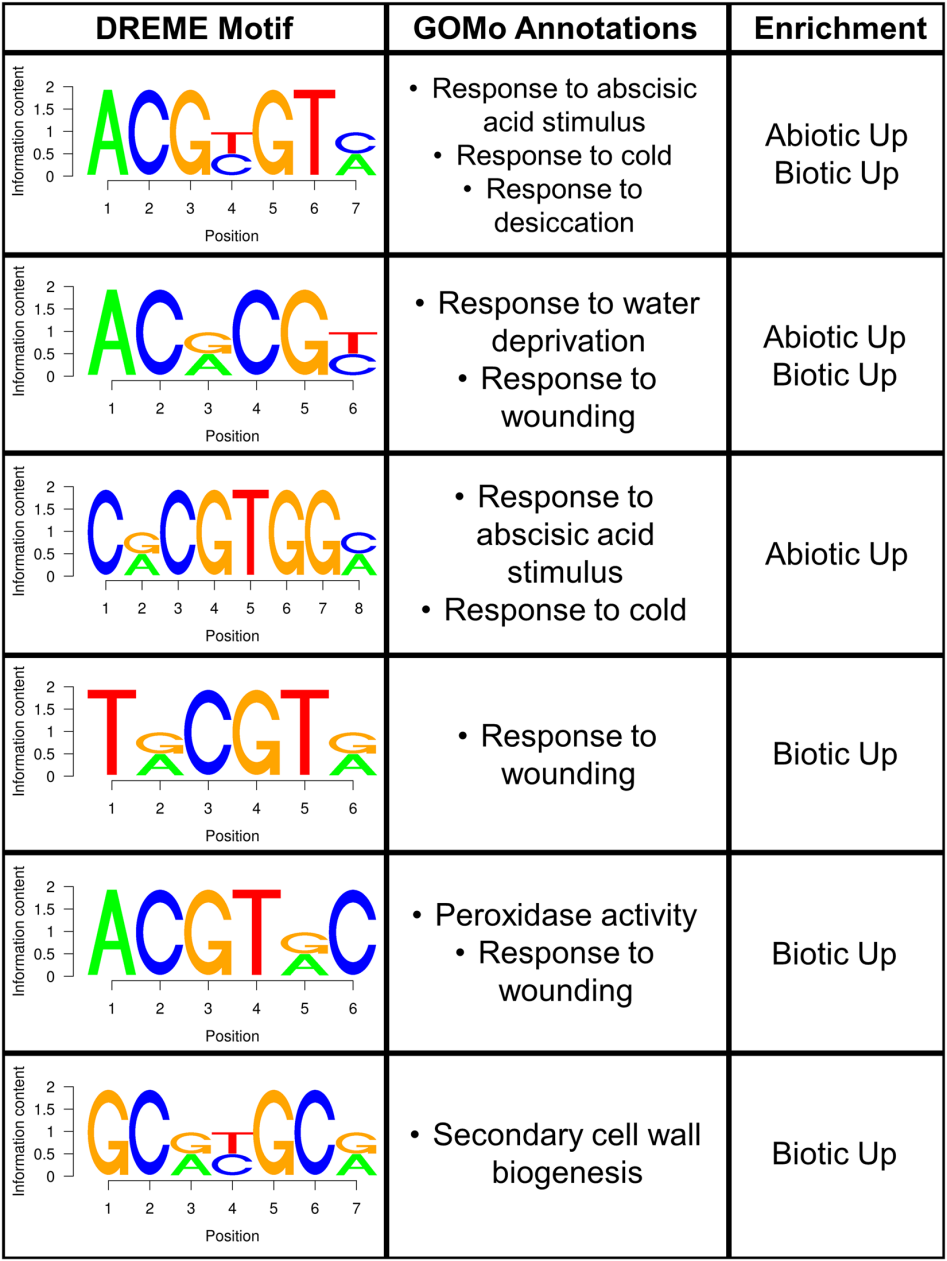
De novo discovered promoter motifs. Sequence logos for motifs discovered via DREME, associated GO term annotations discovered via GOMo, and enrichment within metaDEG sets as determined by Fisher’s exact test (p ≤ 0.05).

We performed d*e novo* promoter motif enrichment analysis to identify potential stress-responsive regulatory elements. There were 22 and 17 motifs discovered in the abiotic and biotic metaDEGs, respectively (Table S6). GO term analysis revealed six motifs that are likely to be involved in stress-responsive pathways (Fig. 4). Many of these motifs contained a sequence similar to the ACGT core sequence of the ABA responsive element (ABRE), an upstream bZIP TF binding sequence^42^, indicating a possible role for bZIP TFs in the core stress response. Of the 21 bZIP TFs we identified as metaDEGs, 17 were up-regulated in response to abiotic stress (Supplementary Data S1), including bZIP23 (MSU: LOC_Os02g52780) and bZIP46 (MSU: LOC_Os06g10880), which are key players in ABA response^43,44^. Biotic stress only up-regulated three bZIP TFs. The enrichment of ABRE-like motifs in the promoters of biotic stress-induced metaDEGs suggests that even though there are fewer bZIP TFs responsive to biotic stresses than abiotic stresses, bZIP TFs may still act as critical regulators of response to biotic stress. One bZIP TF (MSU: LOC_Os08g38020) was a potential node of antagonistic cross-talk, up-regulated by abiotic stress and down-regulated by biotic stress. Taken together, these results indicate that rice utilizes ACGT-bZIP TF to regulate response to both abiotic and biotic stress, and identify bZIP elements as key nodes for further studies.

### Pre-processed publicly available gene expression data validates meta-analysis results

**Figure 5:**
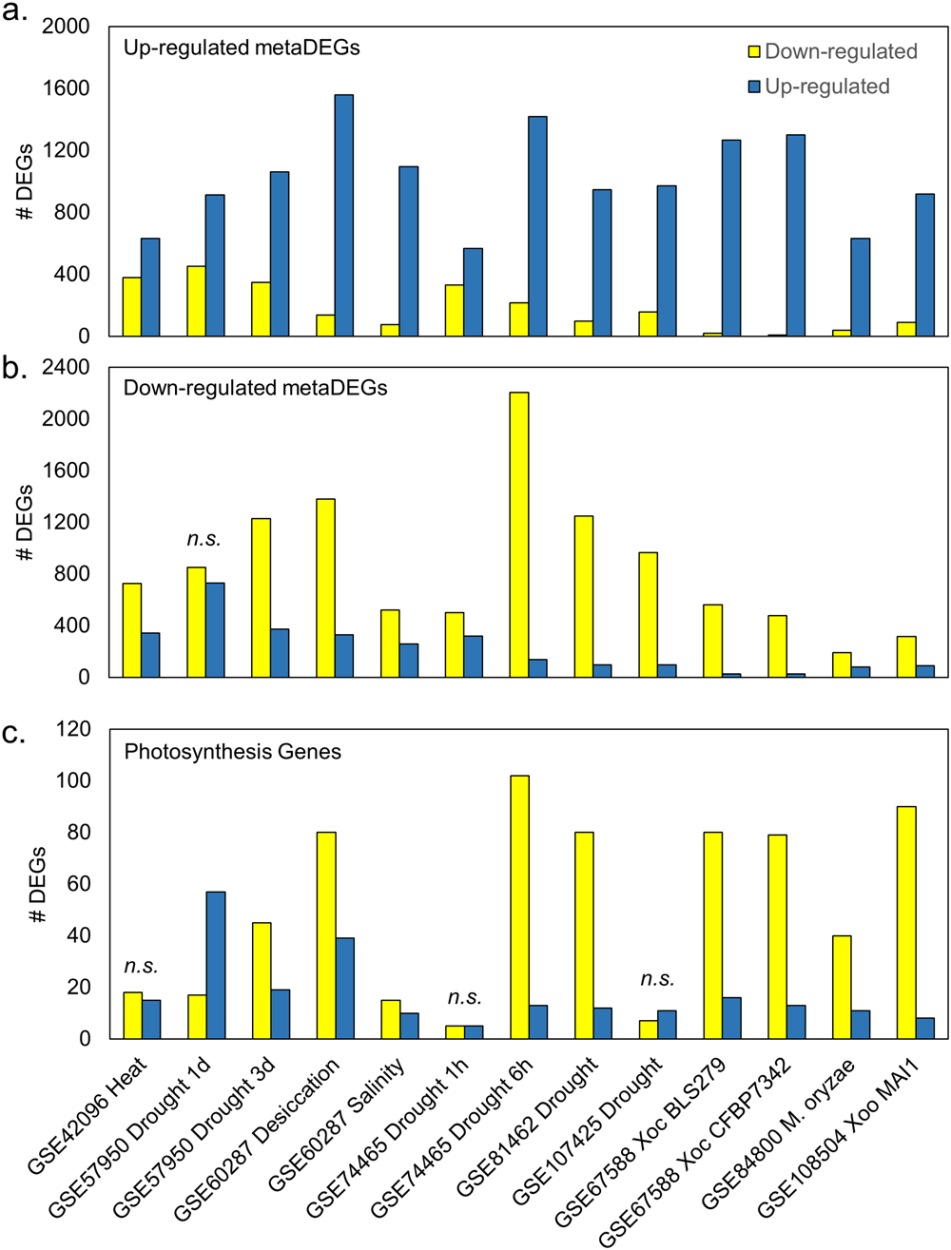
Publicly available gene expression studies validated meta-analysis results. (**a**) Up- and (**b**) Down-regulated metaDEGs and (**c**) photosynthesis-annotated genes generally followed expected trends in pre-processed publicly available gene expression datasets. *n.s*. indicates the counts observed did not differ significantly from counts expected as determined by the χ^2^ goodness of fit test (p > 0.05, see Tables S7 and S8 for all p-values).

To validate the results of the meta-analysis, nine publicly available pre-processed gene expression studies^37,45–52^ were examined for the trends expected from our previous analysis (Table 3). With one exception, all studies fit the expected trends; i.e., up- and down-regulated metaDEGs were more up- and down-regulated than expected by random chance, respectively, as determined by the χ^2^ goodness of fit test (p ≤ 0.05, Fig. 5a,b, Table S7). The study that did not fit the expected trend (GSE57950 drought) had two time-points, with the earlier time-point (1 d after stress) not fitting the expected trend in down-regulated metaDEGs. As in the meta-analysis, photosynthesis genes were mostly down-regulated (Fig. 5c, Table S8). Three studies did not significantly alter photosynthesis gene expression (GSE42096 heat, GSE74465 drought 1 h, GSE107425 drought), and one study up-regulated this pathway (GSE57950 drought 1 d). In the later time-point of the latter study (GSE57950 drought 3d), plants down-regulated photosynthesis-annotated genes, suggesting there may be some temporal effects of drought on altered regulation of photosynthesis, particularly as leaves dehydrate after continued drought. In study GSE108504, rice strongly down-regulated photosynthesis-annotated genes in response to Xoo (Fig. 5c), opposite to the set used in the training data, where these genes were up-regulated by Xoo (Fig. S2). These results validate our meta-analysis approach to finding the rice core stress response.

**Table 3:** NCBI GEO accessions analyzed to validate meta-analysis.

Accession	Stress	Cultivar	Tissue	Time after stress
GSE42096	High temperature	Zhongxian 3037	Leaf	18 d
GSE57950	Drought	Huanghuazhan	Leaf	1 d 3 d
GSE60287	Desiccation Salinity	IR64	Seedling	Unspecified
GSE74465	Drought	Nipponbare	Whole plant	1 h 6 h
GSE81462	Drought	Zhonghua 11	Above-ground	Unspecified
GSE107425	Drought	Zhonghua 11	Shoot	4 d
GSE67588	Xoc BLS279 Xoc CFBP7342	Nipponbare	Leaf	2 d
GSE84800	*M. oryzae* Fr13	Nipponbare	Shoot	4 d
GSE108504	Xoo MAI1	Nipponbare	Leaf	1 d

Xoc indicates *X. oryzae* pv. *oryzicola*; Xoo indicates *X. oryzae* pv. *oryzae*.

## Discussion

A variety of environmental stresses affect plants in the field and can limit crop yield. To endure these stresses, plants respond with coordinated changes to their transcriptome. While these changes are dependent on the specific stress experienced, our results indicate that there is a rice core response to all stresses. With our meta-analysis of publicly available RNA-Seq data of rice experiencing various abiotic and biotic stresses, we identified 5,863 and 2,154 genes that are differentially regulated by abiotic stress and biotic stress, respectively (Fig. 1, Supplementary Data S1). Of these, 913 genes were similarly regulated by both abiotic and biotic stress, while 88 were regulated oppositely (Table S3, Supplementary Data S2). A different study utilized differential expression analysis of rice microarray data to identify genes commonly regulated by abiotic and biotic stresses, and found 40 rice genes that were responsive to both abiotic and biotic stresses^32^. Our meta-analysis of RNA-Seq data identified more of the rice core stress response than this previous comparative microarray analysis. We also validated our meta-analysis approach using additional publicly available studies not used in the training sets; through this validation, we identified sets of stress-responsive genes similar to those found in the meta-analysis (Fig. 5, Tables S7 and S8).

Although the reference genome is annotated with only 45 BP GO terms, we identified several BPs that were altered by stress, including ‘catabolic process’, ‘cell communication’, ‘embryo development’, ‘reproduction’, and ‘response to extracellular stimulus’, which were all up-regulated by stress, and ‘photosynthesis’, ‘protein modification process’, and ‘response to external stimulus’, which were all down-regulated by stress. Photosynthesis is known to be down-regulated by abiotic stresses such as drought, cold, and heat stress^53–56^. This is likely a protective mechanism against plant photooxidative damage during stress^56,57^. In stress tolerant varieties of rice, photosynthetic efficiency is restored, and up-regulation of photosynthesis is physiologically important for yield stability^58,59^. Consistent with these findings, overexpression of a master regulator of photosynthesis enhanced rice tolerance to drought^60^. A range of biotic stresses, including bacterial, viral, and fungal pathogens, also inhibit photosynthesis in plants^61–65^. It is hypothesized that the photosynthesis pathway is a hub of cross-talk in growth and defense trade-offs during plant-pathogen interactions^66^. Studying the roles of the photosynthesis-regulated metaDEGs identified in this study may facilitate the development of stress tolerant varieties of rice.

Various stresses positively induced phytohormone pathways (Fig. 2). Abiotic stress up-regulated genes responsive to ABA, auxin, JA, and SA, while biotic stress up-regulated genes responsive to the same hormones plus cytokinin and ethylene. The ABA, JA, and SA pathways were the most significantly up-regulated hormone pathways in both abiotic and biotic stress. ABA, JA and SA signaling regulate response to abiotic stresses^31,54,55,67^. While JA and SA are positive regulators, ABA tends to be a negative regulator of resistance to pathogens^25,68–70^. ABA is also important to inter-kingdom signaling among pathogens and plants. For example, synthesis of ABA by the fungal pathogen *M. oryzae* during interactions with rice is necessary for pathogen virulence^71^. Plant-synthesized ABA promotes rice susceptibility to the bacterial pathogen *X. oryzae* pv. *oryzae* (Xoo) and even induces swimming in the bacteria^26,72^. While our results show that both ABA and SA are induced during response to biotic stress, ABA-induced susceptibility to Xoo is due to ABA suppressing SA-mediated defense^26^. We previously hypothesized that ABA is a node of cross-talk in the rice response to simultaneous high temperature stress and *X. oryzae* infection^35^. The results from our current study show that cross-talk among ABA, JA and SA response pathways makes the contribution of each hormone to the rice transcriptome unclear (Fig. 3). Notably, ABA-regulated genes appear to dominate the hormone response during stress. That is, of the metaDEGs responsive to JA and SA, most were also responsive to ABA (Fig. 3, Table S5). These intertwined pathways are critical to plant stress responses, which frequently occur simultaneously, emphasizing that additional study of hormonal cross-talk is needed to provide insights into how to improve plant health.

Our results open the path to future avenues of research, including both *in silico* and *in planta* studies. We immediately provide candidate genes for studying multiple stress responses in rice. For example, the prevalence of enriched ABRE-like promoter motifs suggest that the bZIP TFs identified here are good candidate regulators of stress responses (Fig. 4, Supplementary Data S1). Our analysis only used studies with rice plants that were sensitive (susceptible) to the different stresses. Future researchers can expand on this work by analyzing the regulation of metaDEGs in studies with stress-tolerant rice varieties. We only found 88 oppositely regulated metaDEGs between abiotic and biotic stresses, but it is likely that stress tolerance and sensitivity oppositely regulate many more genes. The resources and approach provided with this work will allow for a deeper understanding of rice strategies for overcoming stresses.

We present this work as a proof of concept: meta-analysis of diverse transcriptomic data sets is a valid and robust approach to develop hypotheses for how plants respond to stress in general. It is also possible to expand our approach into other systems. For example, with the wealth of publicly available Arabidopsis transcriptome data, researchers can repeat this analysis to identify candidate regulators of Arabidopsis stress response. In systems with few or no publicly available transcriptome studies, the analysis we describe enables researchers to design transcriptome studies from the ground up to study stress response in their systems. Even while limited by the available rice stress-responsive transcriptome data, with multiple tissue types, host cultivars, and few replicates per treatment (Table 2), real trends were identified, indicating it is possible to design experiments in less well-studied plant systems to use with our approach.

To summarize, publicly available rice transcriptome data were used to identify genes and pathways regulated by abiotic stress, biotic stress, and both stress types. We confirmed that photosynthesis is a generally down-regulated pathway in response to all stress types. We also identified stress-induced plant hormone-responsive genes, particularly genes downstream of ABA, JA and SA. With this work, we provide a list of candidate genes to study for improving rice stress tolerance, and thus yield, in light of environmental stresses. This study provides a valid approach to ask additional questions with respect to how plants respond to stress, including but not limited to (1) how tolerant rice varieties respond to stress and (2) how other plants respond to stress.

## Methods

### RNA-Seq Data Acquisition and Processing

Raw sequence data for all accessions were downloaded from NCBI Sequence Read Archive using the SRA Toolkit (https://github.com/ncbi/sra-tools). Adapter sequences and low quality reads were removed with Trimmomatic v0.36^73^. Reads were mapped to the MSU RGAP 7.0 rice reference genome^74^ with STAR v2.5^75^ and counted using HTSeq v0.9.1^76^.

### Differential Gene Expression and Meta-Analyses

Differential gene expression analyses were conducted using the Bioconductor package edgeR^77,78^. For single analyses, genes were considered differentially expressed if the FDR-adjusted p-values were ≤0.01. For meta-analyses, Fisher’s sum of logs method (as discussed by Rau *et al*.^79^ and implemented in the R package metap v0.8; https://cran.r-project.org/web/packages/metap/index.html) was used to combine unadjusted p-values. The p.adjust function in R^80^ was used to adjust the combined p-values for multiple testing with the ‘fdr’ method. Genes were considered differentially expressed in meta-analyses if the adjusted p-values were ≤0.01 and the absolute value of the median log_2_ fold change for all studies within the analysis was ≥1. GO terms were considered enriched within a metaDEG set if the odds ratio estimates relative to background genes was >1 and the FDR-corrected p-values from Fisher’s exact test were ≤0.01.

### Phytohormone-responsive Gene Analysis

Known hormone-responsive genes were from Garg *et al*.^81^. The chisq.test function in R was used for χ^2^ goodness of fit test, with a p-value threshold of 0.05, to determine if number of hormone-responsive up- and down-regulated genes were as expected due to random chance. For the χ^2^ tests, the expected number of up- and down-regulated genes was proportional to the total number of up- and down-regulated genes in the background set.

### *De novo* Promoter Motif Discovery

Promoter motifs and associated GO terms were discovered with DREME^82^ and GOMo^83^ respectively, using 500 bp regions upstream of putative transcription start sites. Fisher’s exact test with a p-value threshold of 0.01 was used to determine whether motifs were enriched in metaDEG sets.

### Validation with Pre-processed Gene Expression Studies

Pre-processed gene expression studies were acquired from NCBI Gene Expression Omnibus. Because many of these studies lacked replicates, regulatory patterns of genes were estimated by finding the ratio of normalized expression value of treatment to control, disregarding log_2_ fold changes with absolute value < 1. Studies GSE67588 and GSE108504 were normalized by calculating number of gene reads per millions of total reads. The χ^2^ goodness of fit test with a p-value threshold of 0.05 was used to determine whether the counts of up- and down-regulated were as expected by random chance. For the χ^2^ tests, the expected number of up- and down-regulated genes was proportional to the total number of up- and down-regulated genes in the background set.

## Supplementary information

Supplementary Information

Supplementary Data S1

Supplementary Data S2

Supplementary Data S3

## Data Availability

The datasets generated during and/or analyzed during the current study are available from the corresponding author on reasonable request.
