# Supplementary material for: Abiotic and biotic stresses induce a core transcriptome response in rice
Source: Sci Rep. 2019 Apr 18;9:6273. doi: 10.1038/s41598-019-42731-8 (PMC6472405; doi:10.1038/s41598-019-42731-8)
Supplement: Supplementary file 1 — Supplementary Information [file 41598_2019_42731_MOESM1_ESM.pdf]

Supplementary Information

Abiotic and biotic stresses induce a core transcriptome response in rice

Stephen P. Cohen[1,2], Jan E. Leach[1,*]

[1]Department of Bioagricultural Sciences and Pest Management, Colorado State University, CO, United States, 80523-1177

[2]Cell and Molecular Biology Graduate Program, Colorado State University, CO, United States, 80523

*Corresponding author; email: Jan.Leach@colostate.edu

Table S1. Sequence reads and mapping summary statistics for studies used in this analysis.

SRA Accession	SRA File	Raw Reads			Mapped Reads		Mapped to Genes	
		Total	High-quality	%	Total	%	Total	%
SRP004651	SRR074138	2846609	2081083	73.1	1639951	57.6	647117	22.7
	SRR074139	4275874	4098121	95.8	3123652	73.1	1260614	29.5
	SRR074152	2813770	2102466	74.7	1545280	54.9	589689	21.0
	SRR074153	4100071	3965068	96.7	2800270	68.3	1112812	27.1
SRP049040	SRR1615264	17789340	15830450	89.0	15063183	84.7	13399817	75.3
	SRR1615265	18653720	16503925	88.5	15939728	85.5	14567482	78.1
	SRR1615270	20593774	18494227	89.8	17957374	87.2	16293467	79.1
	SRR1615271	22838476	19933545	87.3	19358889	84.8	18026902	78.9
	SRR1615276	22912622	19997866	87.3	19337019	84.4	17859494	77.9
	SRR1615277	22824036	19912550	87.2	19178057	84.0	17560005	76.9
SRP049444	SRR1636849	19092472	17838233	93.4	17531136	91.8	15874146	83.1
	SRR1636850	19230528	18060948	93.9	17732822	92.2	16024851	83.3
	SRR1636851	19145050	17972749	93.9	17568563	91.8	15800857	82.5
	SRR1636852	19204242	18077313	94.1	17655624	91.9	15947097	83.0
SRP052306	SRR1761528	14664192	14336202	97.8	14126322	96.3	12561495	85.7
	SRR1761529	16490701	16079832	97.5	15930835	96.6	14136931	85.7
	SRR1761530	14302686	13957683	97.6	13854692	96.9	12341694	86.3
	SRR1761531	14857078	14499467	97.6	14378412	96.8	12714596	85.6
SRP056884	SRR1952778	24830499	23223823	93.5	22989791	92.6	20741839	83.5
	SRR1952779	4883837	4628841	94.8	4569527	93.6	4173349	85.5
	SRR1952780	20256732	18175744	89.7	18025351	89.0	16268893	80.3
	SRR1952793	42745641	39611721	92.7	39235112	91.8	35101114	82.1
	SRR1952794	21223770	20138835	94.9	19936854	93.9	18145834	85.5
	SRR1952795	23224262	21169649	91.2	20983836	90.4	18960452	81.6
	SRR1952799	11473000	10789043	94.0	10307039	89.8	9175002	80.0
	SRR1952800	7052129	5782355	82.0	5735075	81.3	5189344	73.6
	SRR1952801	94865601	86940356	91.7	85208991	89.8	76480884	80.6
	SRR1952808	26656102	24944374	93.6	24623033	92.4	22051552	82.7

	SRR1952809	11353463	9116423	80.3	9042009	79.6	8204216	72.3
	SRR1952810	21537709	19914067	92.5	19762897	91.8	17545990	81.5
SRP065503	SRR2862232	10881310	10666806	98.0	10476630	96.3	8796680	80.8
	SRR2862233	11549952	11335079	98.1	11184408	96.8	9652944	83.6
	SRR2862234	14669472	14400690	98.2	14196515	96.8	12212313	83.2
	SRR2862235	14835261	13831522	93.2	12759480	86.0	9019246	60.8
	SRR2862236	14492224	13457128	92.9	12427566	85.8	8693814	60.0
	SRR2862237	14732837	13866180	94.1	13034861	88.5	9616513	65.3
SRP071248	SRR3209769	33012246	32544030	98.6	32183678	97.5	28458579	86.2
	SRR3209770	28693929	28272609	98.5	27919697	97.3	24568753	85.6
	SRR3209771	33738285	32592932	96.6	32322917	95.8	28552310	84.6
	SRR3209772	31951497	31491434	98.6	31136484	97.4	27552347	86.2
	SRR3209773	34717753	34209223	98.5	33932453	97.7	29807973	85.9
	SRR3209774	28803587	28402703	98.6	28138752	97.7	24721147	85.8
	SRR3209775	32798250	32317710	98.5	31714289	96.7	27963729	85.3
	SRR3209776	33838322	33335561	98.5	32500089	96.0	28722587	84.9
	SRR3209777	32797025	32257404	98.4	32045115	97.7	28278419	86.2
	SRR3209778	35848188	35304074	98.5	34446373	96.1	30406765	84.8
	SRR3209779	37177413	36585163	98.4	36146494	97.2	31734877	85.4
	SRR3209780	24165701	23716826	98.1	23503601	97.3	20649808	85.5
SRP076382	SRR3657371	22197650	20998530	94.6	20639867	93.0	18214810	82.1
	SRR3657372	22268264	21738274	97.6	21537816	96.7	18805709	84.5
	SRR3657373	22266057	21732275	97.6	21385244	96.0	18725054	84.1
	SRR3657374	64675050	63208865	97.7	61855799	95.6	53979022	83.5
	SRR3657375	64439195	62839529	97.5	61104240	94.8	51675412	80.2
	SRR3657376	64563225	63079703	97.7	61465550	95.2	52331655	81.1
SRP101342	SRR5311338	28856641	28556116	99.0	28045305	97.2	25119042	87.0
	SRR5311339	30695442	30362383	98.9	29840798	97.2	26626693	86.7
	SRR5311340	41141433	40804827	99.2	40082712	97.4	36075724	87.7
	SRR5311341	30123157	29815532	99.0	29102390	96.6	26153865	86.8
SRP113286	SRR5856927	45759126	42925774	93.8	36858231	80.5	33434870	73.1
	SRR5856928	42646908	39885840	93.5	34578376	81.1	31388151	73.6

	SRR5856929	62531390	58566201	93.7	49797889	79.6	45432151	72.7
	SRR5856930	48560300	45410331	93.5	40126712	82.6	36866849	75.9
	SRR5856931	53548032	50082357	93.5	45393291	84.8	41719887	77.9
	SRR5856932	42810176	40099526	93.7	35954083	84.0	32689730	76.4
SRP115030	SRR5909330	30885880	29633997	95.9	28103798	91.0	23947753	77.5
	SRR5909331	43120834	41191189	95.5	38723981	89.8	31260292	72.5
	SRR5909332	34017864	32708190	96.2	30951045	91.0	25933263	76.2
	SRR5909333	25897684	24806086	95.8	22676004	87.6	16798889	64.9
	SRR5909334	31044302	29575566	95.3	28524764	91.9	24513853	79.0
	SRR5909335	32654458	31285854	95.8	30187414	92.4	26092712	79.9

All percentages are per total raw reads per row.

Table S2. Total number of DEGs identified in each study and the amount of DEGs per study retained after meta-analysis.

Stress	# DEGs	# Retained	% Retained
Drought long day	11644	4531	39
Drought short day	11292	4393	39
Drought	9710	3925	40
Salt	7950	2438	31
High Temperature	1220	505	41
Cold	1545	663	43
Xoc BLS256	7625	1194	16
Xoc RS105	3163	807	26
Xoc CFBP7331	2684	754	28
Xoo PXO349 1 dpi	5164	559	11
Xoo PXO349 2 dpi	6477	673	10
M. oryzae ZB13	1465	436	30
M. oryzae Guy11	4756	488	10
Rice Stripe Virus	7620	881	12
Rice Dwarf Virus	8320	1108	13

Xoc indicates *X. oryzae* pv. *oryzicola*; Xoo indicates *X. oryzae* pv. *oryzae*

Table S3. Number and percentage of metaDEGs identified with all possible expression patterns.

Expression Pattern	metaDEGs	Abiotic metaDEGs (%)	Biotic metaDEGs (%)	All metaDEGs (%)
Abiotic Up	1929	32.9	0	27.5
Abiotic Up Biotic Up	615	10.5	28.6	8.8
Abiotic Up Biotic Down	18	0.3	0.8	0.3
Abiotic Down	2933	50.0	0	41.8
Abiotic Down Biotic Up	70	1.2	3.2	1.0
Abiotic Down Biotic Down	298	5.1	13.8	4.2
Biotic Up	753	0	35.0	10.7
Biotic Down	400	0	18.6	5.7

Table S4. Biological process GO terms significantly enriched within metaDEG sets.

Abiotic Up-regulated<br>GO Term	# in pattern	# in background	p-value*
biological process	797	10051	7.60E-29
biosynthetic process	557	5922	3.01E-35
carbohydrate metabolic process	152	1287	2.46E-16
catabolic process	196	1812	3.59E-17
cell communication	34	326	0.001511
cellular homeostasis	35	308	0.000287
cellular process	761	9396	8.09E-30
embryo development	70	740	7.43E-05
lipid metabolic process	140	1236	7.11E-14
metabolic process	863	9239	2.41E-58
multicellular organismal development	177	1861	3.66E-11
nucleobase, nucleoside, nucleotide and nucleic acid metabolic process	377	4604	3.29E-14
pollen-pistil interaction	14	104	0.006178
post-embryonic development	117	1456	0.000158
reproduction	106	1092	2.00E-07
response to abiotic stimulus	322	2703	3.01E-35
response to biotic stimulus	133	1272	5.04E-11
response to endogenous stimulus	198	1818	1.20E-17
response to extracellular stimulus	37	356	0.001101
response to stress	466	4198	7.52E-45
secondary metabolic process	52	531	0.000335
signal transduction	143	1810	6.36E-05
transport	283	2997	2.88E-17
**Abiotic Down-regulated**<br>**GO Term**	**# in pattern**	**# in background**	**p-value***
anatomical structure morphogenesis	145	996	1.75E-13
biological process	906	9942	3.07E-15

biosynthetic process	629	5850	7.77E-24
carbohydrate metabolic process	149	1290	1.47E-07
cell differentiation	77	634	4.64E-05
cell growth	85	482	5.00E-12
cellular component organization	197	1739	3.81E-09
cellular homeostasis	48	295	2.13E-06
cellular process	1076	9081	1.11E-64
generation of precursor metabolites and energy	115	365	2.63E-33
lipid metabolic process	173	1203	1.57E-15
metabolic process	1275	8827	8.80E-139
multicellular organismal development	187	1851	1.06E-05
nucleobase, nucleoside, nucleotide and nucleic acid metabolic process	405	4576	1.71E-05
photosynthesis	120	204	1.76E-56
pollen-pistil interaction	19	99	0.000463
post-embryonic development	142	1431	0.000285
protein modification process	456	3522	1.93E-31
response to abiotic stimulus	377	2648	7.78E-33
response to biotic stimulus	174	1231	4.58E-15
response to endogenous stimulus	226	1790	2.16E-14
response to external stimulus	33	147	2.08E-07
response to stress	455	4209	3.30E-17
ripening	3	3	0.007739
secondary metabolic process	79	504	3.76E-09
signal transduction	245	1708	1.47E-21
transport	353	2927	1.87E-19
tropism	28	103	6.94E-08
**Biotic Up-regulated**			
**GO Term**	**# in pattern**	**# in background**	**p-value***
biological process	391	10457	3.33E-06
biosynthetic process	299	6180	6.03E-16

catabolic process	83	1925	0.00306
cell death	27	451	0.00351
cellular process	374	9783	8.00E-07
generation of precursor metabolites and energy	27	453	0.00351
lipid metabolic process	85	1291	6.46E-10
metabolic process	500	9602	3.54E-37
multicellular organismal development	92	1946	9.60E-05
nucleobase, nucleoside, nucleotide and nucleic acid metabolic process	219	4762	1.94E-09
pollen-pistil interaction	10	108	0.005523
post-embryonic development	74	1499	0.000162
reproduction	54	1144	0.003885
response to abiotic stimulus	181	2844	1.49E-19
response to biotic stimulus	119	1286	2.86E-24
response to endogenous stimulus	146	1870	4.65E-23
response to extracellular stimulus	25	368	0.001114
response to stress	310	4354	1.70E-43
secondary metabolic process	46	537	8.11E-09
signal transduction	90	1863	6.89E-05

**Biotic Down-regulated**<br>**GO Term**	**# in pattern**	**# in background**	**p-value***
biological process	193	10655	0.00389
biosynthetic process	124	6355	0.003655
carbohydrate metabolic process	48	1391	1.19E-06
cellular process	198	9959	2.09E-05
generation of precursor metabolites and energy	21	459	6.43E-05
metabolic process	221	9881	3.51E-10
photosynthesis	29	295	3.55E-13
response to biotic stimulus	41	1364	0.00018
response to external stimulus	10	170	0.001686
response to stress	94	4570	0.003655

*p-values are calculated with Fisher’s exact test and corrected using the FDR method

Table S5. Number of observed and expected phytohormone-induced metaDEGs.

Abiotic metaDEGs					
**Pathway**	**Up-regulated**	**Expected Up-regulated**	**Down-regulated**	**Expected Down-regulated**	**p-value***
**ABA**	765	351	38	452	1.1E-190
**auxin**	52	35	28	45	1.3E-04
**cytokinin**	39	33	36	42	1.6E-01
**ethylene**	21	16	15	20	9.4E-02
**JA**	85	56	44	73	2.6E-07
**SA**	184	90	23	117	1.2E-39
**JA‡**	21	11	5	15	7.2E-05
**SA‡**	34	16	2	20	1.6E-09

Biotic metaDEGs					
**Pathway**	**Up-regulated**	**Expected Up-regulated**	**Down-regulated**	**Expected Down-regulated**	**p-value***
**ABA**	239	165	8	82	1.5E-23
**auxin**	53	38	4	19	2.5E-05
**cytokinin**	32	23	3	12	1.4E-03
**ethylene**	26	19	2	9	4.6E-03
**JA**	93	64	3	32	3.4E-10
**SA**	139	95	3	47	4.3E-15
**JA‡**	40	29	4	15	4.7E-04
**SA‡**	45	31	2	16	1.6E-05

*p-values are calculated with χ^2 goodness of fit test; ‡ indicates genes not responsive to ABA

Table S6. All *de novo* discovered promoter motifs.

Motif	Discovered In	Enriched In*			
		Abiotic Up	Abiotic Down	Biotic Up	Biotic Down
ACGYGTM	Abiotic Up	Yes	No	Yes	No
TRCGTR	Abiotic Up	Yes	Yes	Yes	No
CTATAWA	Abiotic Up	Yes	Yes	Yes	Yes
CRCGTGGM	Abiotic Up	Yes	No	No	No
AAAAADA	Abiotic Up	Yes	Yes	Yes	Yes
AGTASTA	Abiotic Up	Yes	Yes	Yes	Yes
AAACG	Abiotic Up	Yes	Yes	Yes	No
CACGNCAC	Abiotic Up	Yes	No	Yes	No
DAAAAAAH	Abiotic Down	Yes	Yes	Yes	Yes
TAGCTR	Abiotic Down	Yes	Yes	Yes	Yes
AMTRTA	Abiotic Down	Yes	Yes	No	Yes
AATTW	Abiotic Down	Yes	Yes	Yes	Yes
MTGMAA	Abiotic Down	Yes	Yes	Yes	Yes
STAGTA	Abiotic Down	Yes	Yes	Yes	No
TKCAGW	Abiotic Down	Yes	Yes	Yes	Yes
DCCACACA	Abiotic Down	No	Yes	Yes	No
TAYATR	Abiotic Down	Yes	Yes	Yes	Yes
ATGTKW	Abiotic Down	No	Yes	No	Yes
ACKTACG	Abiotic Down	Yes	Yes	Yes	Yes
AYGMATG	Abiotic Down	Yes	Yes	Yes	Yes
AAAT	Abiotic Down	Yes	Yes	Yes	Yes
CAGYA	Abiotic Down	Yes	Yes	Yes	Yes
ACGTRC	Biotic Up	Yes	Yes	Yes	No
ACRCGY	Biotic Up	Yes	No	Yes	No
GCRYGCR	Biotic Up	Yes	Yes	Yes	No
SCTATAWA	Biotic Up	Yes	Yes	Yes	Yes
CGATCRW	Biotic Up	Yes	No	Yes	No
YAGCTR	Biotic Up	Yes	Yes	Yes	Yes
GTTTGAM	Biotic Up	Yes	No	Yes	No
AGTASTAB	Biotic Up	Yes	Yes	Yes	No
TGCABA	Biotic Down	No	Yes	No	Yes
AGCTASY	Biotic Down	Yes	Yes	Yes	Yes

ADAAAAA	Biotic Down	Yes	Yes	Yes	Yes
ATAWATA	Biotic Down	Yes	Yes	Yes	Yes
TGCAW	Biotic Down	Yes	Yes	No	Yes
CWCACW	Biotic Down	Yes	Yes	Yes	Yes
CAGTD	Biotic Down	No	Yes	No	Yes
ATWTA	Biotic Down	Yes	Yes	Yes	Yes
CATYTTGC	Biotic Down	No	No	No	Yes

*Enrichment determined by Fisher's exact test ($p \leq 0.05$)

Table S7. Number of metaDEGs up- and down-regulated within pre-processed gene expression studies.

Study	Up-regulated metaDEGs	Expected Up-regulated	Down-regulated metaDEGs	Expected Down-regulated	p-value*
**Abiotic Stress Up-regulated metaDEGs**					
**GSE42096**	632	501	380	511	1.8E-16
**GSE57950 1 d**	914	643	453	724	8.0E-49
**GSE57950 3 d**	1062	620	349	791	3.0E-124
**GSE60287 dess.**	1557	929	139	767	3.8E-206
**GSE60287 salinity**	1095	697	77	475	5.8E-124
**GSE74465 1 h**	567	404	331	494	7.8E-28
**GSE74465 6 h**	1417	504	217	1130	0
**GSE81462**	945	405	98	638	5.9E-258
**GSE107425**	973	431	157	699	1.3E-241
**Abiotic Stress Down-regulated metaDEGs**					
**Study**	**Up-regulated metaDEGs**	**Expected Up-regulated**	**Down-regulated metaDEGs**	**Expected Down-regulated**	**p-value***
**GSE42096**	342	530	728	540	1.4E-30
**GSE57950 1 d**	729	743	850	836	0.48
**GSE57950 3 d**	374	705	1230	899	2.9E-62
**GSE60287 dess.**	328	937	1381	772	1.4E-192
**GSE60287 salinity**	257	463	522	316	4.6E-51
**GSE74465 1 h**	319	369	500	450	4.5E-4
**GSE74465 6 h**	136	722	2206	1620	1.5E-151
**GSE81462**	97	523	1250	824	2.3E-125
**GSE107425**	97	405	965	657	2.5E-84
**Biotic Stress Up-regulated metaDEGs**					
**Study**	**Up-regulated metaDEGs**	**Expected Up-regulated**	**Down-regulated metaDEGs**	**Expected Down-regulated**	**p-value***
**GSE67588 BLS279**	1267	804	20	483	1.6E-156
**GSE67588 CFBP7342**	1300	874	10	436	1.1E-137
**GSE84800**	632	465	40	207	3.0E-44
**GSE108504**	918	576	90	432	4.7E-105
**Biotic Stress Down-regulated metaDEGs**					
**Study**	**Up-regulated metaDEGs**	**Expected Up-regulated**	**Down-regulated metaDEGs**	**Expected Down-regulated**	**p-value***
**GSE67588 BLS279**	25	367	562	220	6.1E-187
**GSE67588 CFBP7342**	25	335	478	168	5.7E-190
**GSE84800**	81	188	190	83	3.7E-45
**GSE108504**	90	232	316	174	5.1E-46

*p-values are calculated with χ^2 goodness of fit test; dess. = dessication

Table S8. Number of photosynthesis-annotated genes differentially regulated in pre-processed gene expression studies.

Study	Up-regulated	Expected Up-regulated	Down-regulated	Expected Down-regulated	p-value*
**GSE42096**	15	16	18	17	0.73
**GSE57950 1 d**	57	35	17	39	3.0E-7
**GSE57950 3 d**	19	28	45	36	0.023
**GSE60287 dess.**	39	65	80	54	1.7E-6
**GSE60287 salinity**	10	15	15	10	0.041
**GSE74465 1 h**	5	5	5	5	1
**GSE74465 6 h**	13	35	102	80	8.3E-6
**GSE81462**	12	36	80	56	2.9E-7
**GSE107425**	11	7	7	11	0.053
**GSE67588 BLS279**	16	60	80	36	1.8E-20
**GSE67588 CFBP7342**	13	61	79	31	3.4E-26
**GSE84800**	11	35	40	16	4.4E-13
**GSE108504**	8	56	90	42	1.1E-22

*p-values are calculated with χ^2 goodness of fit test; dess. = dessication

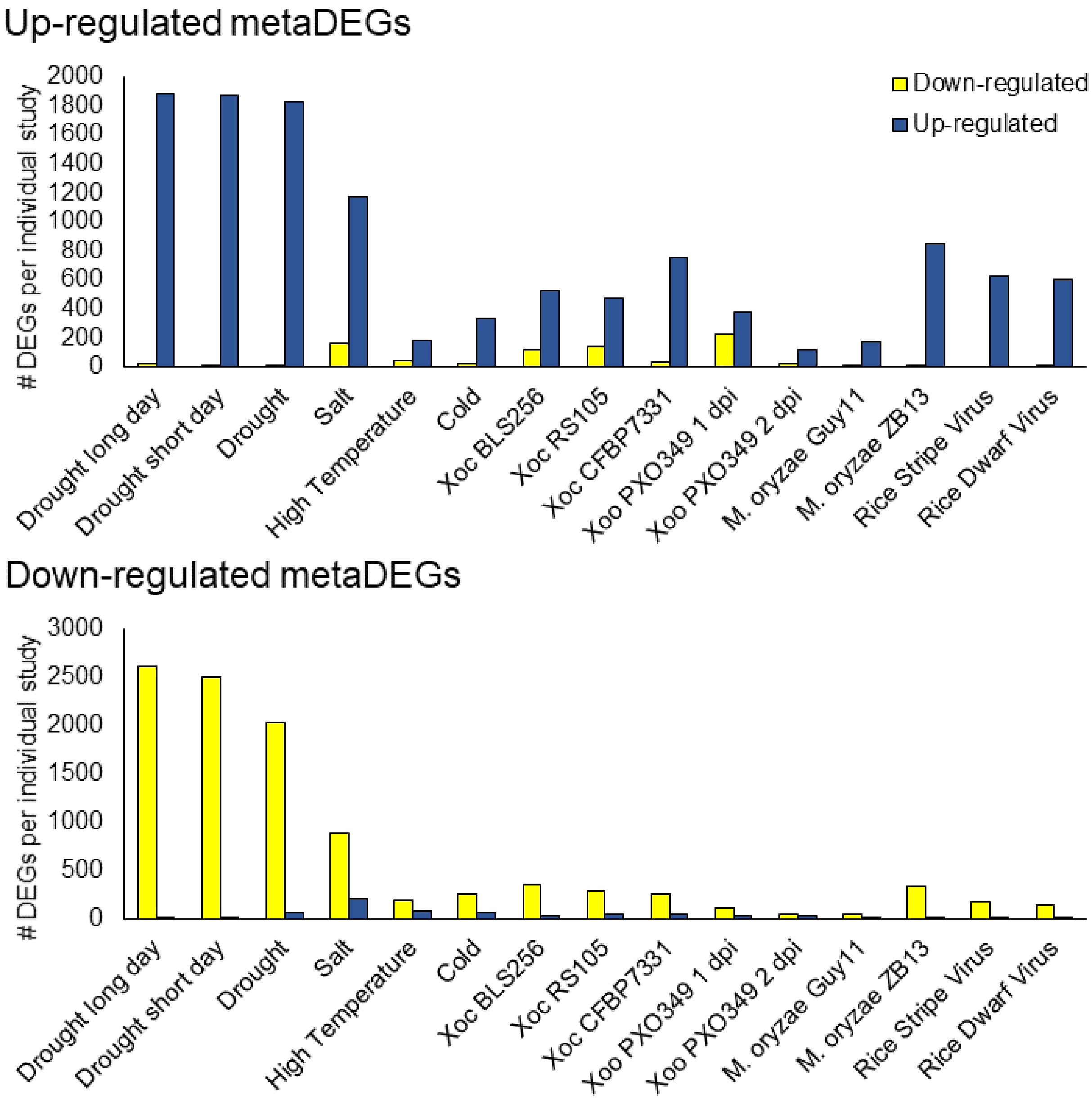

Supplementary Fig. S1. DEGs per study retained in meta-analysis followed the expected regulatory trends. The DEGs retained as up-regulated (top) and down-regulated (bottom) metaDEGs were mostly up- and down-regulated, respectively, within each individual study.

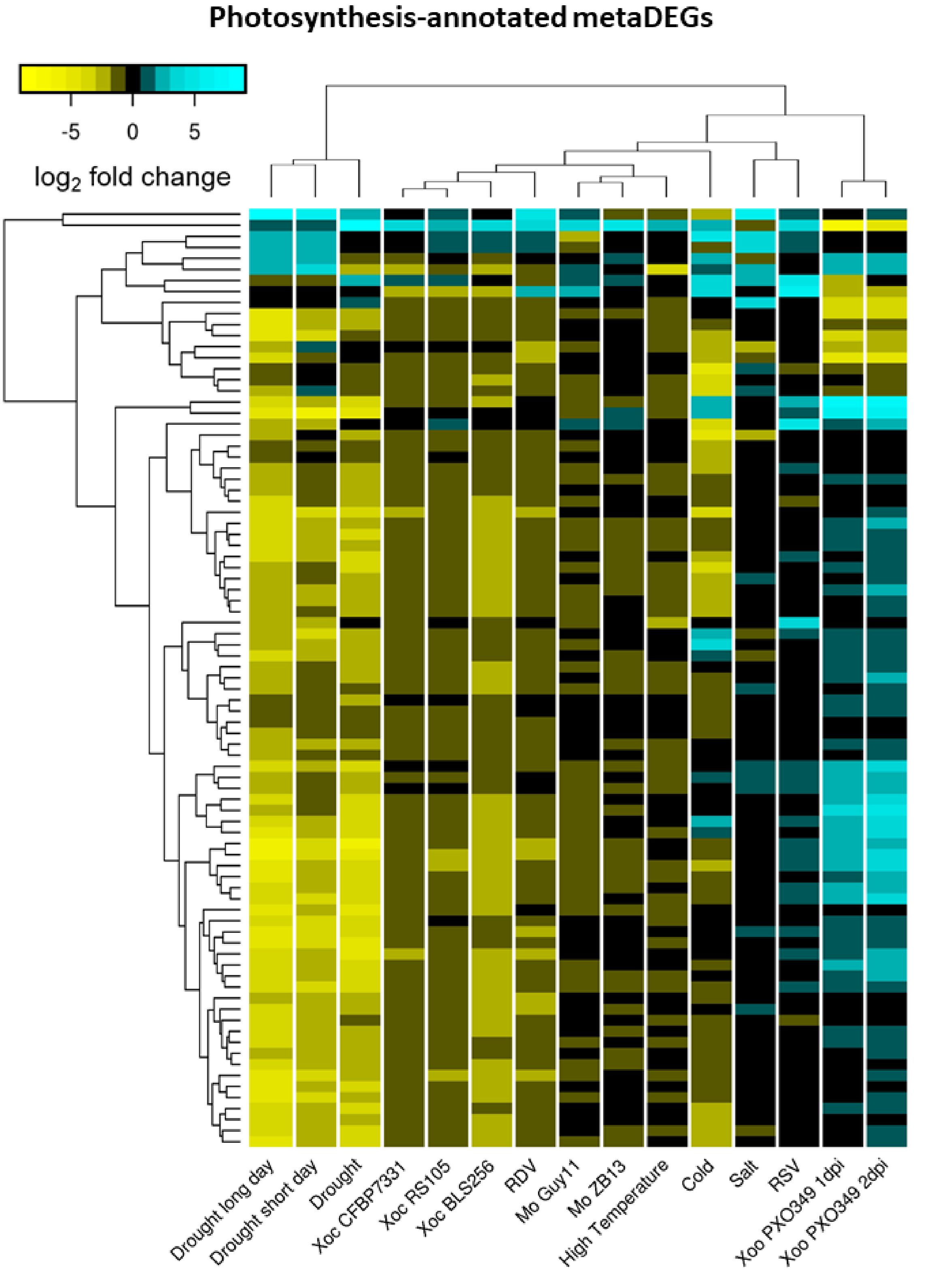

Supplementary Fig. S2. Stress down-regulated photosynthesis in rice. Gene expression ($\log_2$ fold changes) for stresses relative to controls (columns) are shown for photosynthesis-annotated metaDEGs (rows).
